# Anterior Cruciate Ligament (ACL) Reconstruction With Lateral Meniscal Repair With and Without Lateral Extra-articular Tenodesis: A Prospective Comparative Study of Functional and Rotational Outcomes

**DOI:** 10.7759/cureus.116356

**Published:** 2026-09-16

**Authors:** Jerin George, Joseph Sajeev, Mevin M Nedumparampil, Rajkumar Amaravathi

**Affiliations:** 1 Orthopaedics, St. John's Medical College Hospital, Bangalore, IND; 2 Orthopaedic Surgery, St. John's Medical College Hospital, Bangalore, IND

**Keywords:** anterior cruciate ligament reconstruction, lateral extra-articular tenodesis, meniscal repair, modified lemaire technique, pivot shift test, rotational stability

## Abstract

Background

Residual anterolateral rotatory instability (ALRI) following anterior cruciate ligament (ACL) reconstruction (ACLR) may adversely affect functional outcomes and graft stability. Lateral extra-articular tenodesis (LET) has been proposed as an adjunct to ACLR to improve rotational stability. This study aimed to compare functional and rotational outcomes following ACLR with lateral meniscal repair with or without LET.

Methods

This prospective comparative study included 40 patients with ACL injury and concomitant lateral meniscal tear. Patients were allocated to Group A (ACLR + lateral meniscal repair + LET; n = 20) or Group B (ACLR + lateral meniscal repair; n = 20). Allocation was non-randomized and based on clinical risk factors, with patients demonstrating greater rotational instability preferentially allocated to LET. Functional outcomes were assessed using the International Knee Documentation Committee (IKDC) rating, Knee Injury and Osteoarthritis Outcome Score (KOOS), and Lysholm score. Rotational stability was assessed using the pivot-shift test at 12 months. Within- and between-group comparisons were performed using paired- and independent-sample statistical tests, as appropriate.

Results

Both groups demonstrated significant postoperative improvement in functional outcomes. At 12 months, mean IKDC scores were 84.8 ± 7.6 in Group A and 76.1 ± 8.6 in Group B, while mean KOOS scores were 88.8 ± 4.7 and 81.6 ± 6.4, respectively (both *P* < 0.05). Rotational stability was better in Group A, with 85% of patients demonstrating grade 0 pivot shift compared with 15% in Group B (*P* < 0.001). The LET group had greater baseline rotational instability, reflecting the indication-based treatment allocation. The mean 12-month Visual Analogue Scale (VAS) score was higher in Group A than Group B (1.9 vs 0.8).

Conclusion

In this cohort of patients undergoing ACLR with lateral meniscal repair, the addition of LET was associated with improved postoperative rotational stability and functional outcomes. However, the non-randomized, indication-based allocation and baseline differences in rotational instability introduce potential confounding by indication. Therefore, these findings should not be interpreted as definitive evidence of a causal treatment effect. Larger, adequately powered prospective studies with standardized graft selection and balanced baseline characteristics are required to confirm the independent effect of LET.

## Introduction

Anterior cruciate ligament (ACL) injuries are among the most frequent knee injuries in orthopaedic practice; in the United States, there are over 200,000 ACL injuries and 100,000 ACL reconstructions (ACLRs) each year [1]. Restoration of knee stability and function following ACL injury remains a key goal of surgical management [2].

Anterolateral rotatory instability (ALRI), which is usually caused by ACL injuries, is clinically evaluated using the pivot shift test. Even after anatomically performed repairs, some patients still exhibit residual rotational instability, despite the goal of contemporary arthroscopic ACLR techniques being to restore native ligament architecture and biomechanics. Persistent ALRI has been associated with inferior functional outcomes and increased risk of graft failure [3]. Biomechanical studies have demonstrated that the anterolateral complex acts as a secondary stabilizer to the ACL, contributing significantly to the control of tibial internal rotation and rotatory knee stability [4]. Increasing attention has therefore been directed toward addressing these structures during ACL reconstruction [5].

Lateral extra-articular tenodesis (LET) procedures aim to increase rotational stability by reinforcing the anterolateral structures of the knee. The modified Lemaire technique is one of the most used LET methods [6]. In this procedure, a strip of the iliotibial band is passed deep to the fibular collateral ligament and fixed proximal and posterior to the lateral femoral epicondyle, thereby improving control of internal tibial rotation [7]. Biomechanical and clinical studies have suggested that combining LET with ACL reconstruction may improve rotational stability and reduce graft strain [8]. However, the clinical benefits of this combined procedure remain a subject of ongoing investigation.

This study aimed to compare the functional outcomes of ACL reconstruction with lateral meniscal repair performed with and without LET using the modified Lemaire technique in patients presenting with grade II pivot shift.

## Materials and methods

This was a prospective comparative study conducted at St. John's Medical College Hospital, Bangalore, Karnataka, India, between May 2023 and September 2024. The study was approved by the Institutional Ethics Committee of St. John's Medical College Hospital (reference number: TH-111/2023), and informed consent was obtained from all participants before enrollment.

Study population

Inclusion Criteria

Inclusion criteria were: Clinical and radiological diagnosis of ACL tears in patients aged 18-59 years; concomitant lateral meniscal tear confirmed on MRI and intraoperatively; patients undergoing primary arthroscopic ACL reconstruction with lateral meniscal repair; and willingness to participate and comply with the follow-up protocol

Exclusion Criteria

Exclusion criteria were: Previous surgery on the affected knee (revision ACLR); multiligament knee injuries (e.g., associated posterior cruciate ligament (PCL), medial collateral ligament (MCL) grade III, or lateral collateral ligament (LCL) injuries); associated medial meniscus root tears or irreparable meniscal injuries requiring meniscectomy; advanced osteoarthritis (Kellgren-Lawrence grade ≥3); skeletally immature patients; concomitant fractures around the knee (except Segond fracture); systemic inflammatory disease or neuromuscular disorders affecting knee function; and Inability to complete the rehabilitation protocol.

Group Allocation

According to the type of surgery, patients were divided into two equal-sized groups. Group allocation in this study was non-randomized. Patients with established high-risk features for graft failure were preferentially assigned to the LET group (Group A), which therefore inherently represented a cohort with greater baseline rotational instability. These risk parameters included having a Segond fracture, playing pivoting sports, having a high level of sporting activity (Tegner activity score ≥7), and having a high-grade pivot shift (grade II or III). Accordingly, Group A comprised patients who underwent ACLR with lateral meniscal repair and additional LET, while Group B included patients who underwent ACLR with lateral meniscal repair without LET.

Sample Size Calculation

The formula for comparing two independent means was used to determine the sample size: $n = \frac{2(Z_\alpha + Z_\beta)^2 \sigma^2}{d^2}$

Z_α_ = 1.96 for the 95% confidence interval, Z_β_ = 0.84 for 80% power, σ = the functional outcome score standard deviation (assumed from prior research ≈ 10), and d = the expected mean difference between groups (assumed ≈ 6 points). Substituting these values yielded a minimum sample size of approximately 40 patients per group (80 patients in total).

The required sample size was calculated based on the anticipated difference in the primary outcome between the two groups, with a two-sided significance level of 5% and 80% statistical power, resulting in a total of 80 patients. However, during the study period, only 40 eligible patients were enrolled, with 20 patients allocated to each group. Therefore, the final sample size was lower than the initially calculated requirement. The study should consequently be considered an exploratory study with limited statistical power for detecting between-group differences, particularly for secondary outcomes and subgroup comparisons.

Surgical technique

To guarantee consistency in technique and reduce inter-operator variability, every surgical procedure was carried out arthroscopically by the same skilled surgical team. The same senior arthroscopic surgeon carried out each procedure arthroscopically. The modified Lemaire technique was used in Group A to perform LET in addition to standard ACLR and meniscus repair. A strip of iliotibial band was passed deep to the fibular collateral ligament and fixed to the lateral femur proximal and posterior to the lateral epicondyle using a 7x25 mm interference screw with the knee at 30° flexion and neutral rotation.

Standard ACLR utilizing autografts was carried out in Group B using standard tibial and femoral tunnels and autografts such as hamstring tendon grafts, bone-patellar tendon-bone grafts, or peroneus longus grafts. An endobutton was used for femoral fixation, and a beta-tricalcium phosphate (TCP) interference screw with the proper diameter was used for tibial fixation (Healthium Medtech, Bengaluru, Karnataka, India). Meniscal repair was carried out using the FAST-FIX Meniscal Repair System (Smith & Nephew plc, Watford, United Kingdom) and an all-inside approach. For bucket-handle tears, a hybrid approach was used: anterior and middle portions were repaired using inside-out sutures with 2-0 FiberWire (Arthrex, Naples, Florida, United States), while the posterior segment was repaired using the all-inside technique.

Rehabilitation protocol

Every patient adhered to a set rehabilitation regimen. Early range of motion exercises were initiated within the first postoperative week. Weight-bearing was allowed as tolerated with brace support for four to six weeks. At six weeks, strengthening exercises were started, and after six months, sports-specific training was gradually resumed. Based on a functional evaluation, a return to pivoting sports was permitted after nine to twelve months.

Clinical evaluation was performed using the International Knee Documentation Committee (IKDC) rating [9], the Knee Injury and Osteoarthritis Outcome Score (KOOS) [10], the Lysholm score [11], and the Visual Analogue Scale (VAS) pain score [12], while stability was assessed using the Lachman test [13], anterior drawer test [14], and pivot shift test [15]. Functional assessment included single-leg and double-leg hop tests [16], although these were recorded but not subjected to statistical analysis. Every patient was assessed prior to surgery as well as six weeks, three months, six months, and 12 months after the procedure.

Statistical analysis

Although multiple outcome measures were recorded, statistical comparison was primarily focused on IKDC, KOOS, and pivot shift outcomes. Data were presented as mean ± standard deviation (SD). Paired t-tests were employed for within-group comparisons and independent t-tests for between-group comparisons. Statistical significance was defined as a p-value of less than 0.05. IBM SPSS Statistics for Windows, version 25.0 (IBM Corp., Armonk, New York, United States) was used to conduct statistical analysis.

## Results

The two groups were broadly similar in demographic characteristics; however, clinically relevant differences were observed in baseline knee stability (Table 1). The LET group had greater preoperative rotational instability, with a higher proportion of patients demonstrating high-grade pivot-shift findings. Differences were also observed in baseline Lachman findings. These baseline differences reflect the indication-based allocation of patients to LET and should be considered when interpreting the postoperative between-group comparisons. 

**Table 1:** Sociodemographic and clinical baseline characteristics of the two groups LET: lateral extra-articular tenodesis

Parameter		Group A (With LET) (n=20)	Group B (Without LET) (n=20)
Age (years), mean±SD		26.20±6.74	33.50±12.03
Height (cm), mean±SD		168.30±6.69	167.90±6.29
BMI, mean±SD		25.62±2.57	25.73±2.33
Duration of symptoms (months), mean±SD		6.80±4.11	6.125±3.15
Sex, n (%)	Male	15 (75%)	13 (65%)
	Female	5 (25%)	7 (35%)
Side affected, n (%)	Left	8 (40%)	7 (35%)
	Right	12 (60%)	13 (65%)
Symptoms, n (%)	Pain	19 (95%)	16 (80%)
	Locking	9 (45%)	13 (65%)
	Instability	20 (100%)	12 (60%)
Mechanism of injury, n (%)	Twisting injury	11 (55%)	9 (45%)
	Blunt injury	9 (45%)	11 (55%)
	Sports-related	7 (35%)	5 (25%)
Graft type, n (%)	Semitendinosus + gracilis	10 (50%)	11 (55%)
	Bone–patellar tendon–bone (BPTB)	2 (10%)	0 (0%)
	Peroneus longus	8 (40%)	8 (40%)
Associated injuries, n (%)	Medial meniscal tear	7 (35%)	4 (20%)

The parameters among various assessment tools in patients with and without LET are summarized in Table 2. At 12 months, Group A demonstrated significantly better functional outcomes compared with Group B. IKDC scores improved to 84.84 ± 7.64 in Group A compared with 76.16 ± 8.67 in Group B (independent samples t-test, t = 3.21, p = 0.002). KOOS scores improved to 88.8 ± 4.7 in Group A compared with 81.6 ± 6.44 in Group B (independent samples t-test, t = 3.89, p < 0.001), and Lysholm scores were 93.65 ± 6.08 in Group A and 86.70 ± 7.69 in Group B (independent samples t-test, t = 3.05, p = 0.004). VAS scores decreased significantly in both groups, indicating acceptable postoperative pain control (paired t-test, p < 0.001 for both groups).

**Table 2:** Functional outcome scores P<0.05 is considered significant; *Independent t test NS: not significant; IKDC: International Knee Documentation Committee; KOOS: Knee Injury and Osteoarthritis Outcome Score; VAS: Visual Analogue Scale

Assessment Tool	Time Point	Group A (n=20), mean±SD	Group B (n=20), mean±SD	Test statistic*	P-value
IKDC Score	Preoperative	62.65±6.32	58.44±7.55	1.91	NS
	3 months	66.92±7.24	66.14±8.33	0.32	NS
	6 months	77.91±7.33	70.01±3.32	4.39	<0.05
	12 months	84.84±7.64	76.16±8.67	3.36	<0.01
KOOS Score	Preoperative	62.65±6.09	62.30±6.74	0.17	NS
	3 months	67.15±5.59	65.95±8.29	0.54	NS
	6 months	78.30±7.26	72.60±7.05	2.52	<0.05
	12 months	88.80±4.70	81.60±6.44	4.04	<0.01
Lysholm Score	3 months	64.64±4.94	66.20±4.95	−1.00	NS
	6 months	80.20±9.30	79.45±7.56	0.28	NS
	12 months	93.65±6.08	86.70±7.69	3.17	<0.01
VAS Score	3 months	4.85±1.42	5.21±1.20	−0.87	NS
	6 months	2.90±1.02	3.10±1.02	−0.62	NS
	12 months	1.90±1.02	0.80±1.01	3.43	<0.05

The parameters of the anterior drawer test among patients with and without LET are presented in Table 3. Clinical stability testing showed comparable results for anterior drawer and Lachman tests in both groups (chi-square test, p > 0.05). However, the pivot shift test demonstrated significantly better rotational stability in Group A, with 85% of patients achieving grade 0 pivot shift compared with 15% in Group B (chi-square test, χ² = 16.2, p < 0.001).

**Table 3:** Clinical examination findings and stability tests P<0.05 considered significant; *chi-square test NS: not significant; LET: lateral extra-articular tenodesis

Parameter	Time point	Grade	Group A (With LET), n (%)	Group B (Without LET), n (%)	χ²*	P-value
Anterior Drawer Test	Preoperative	Grade 2	5 (25%)	5 (25%)	0.00	NS
Anterior Drawer Test	Preoperative	Grade 3	15 (75%)	15 (75%)		
Anterior Drawer Test	Postoperative (12 months)	Grade 0	15 (75%)	16 (80%)	0.14	NS
Anterior Drawer Test	Postoperative (12 months)	Grade 1	5 (25%)	4 (20%)		
Lachman Test	Preoperative	Grade 2	4 (20%)	16 (80%)	14.40	<0.001
Lachman Test	Preoperative	Grade 3	16 (80%)	4 (20%)		
Lachman Test	Postoperative (12 months)	Grade 0	15 (75%)	15 (75%)	0.00	NS
Lachman Test	Postoperative (12 months)	Grade 1	5 (25%)	5 (25%)		
Pivot Shift Test	Preoperative	Grade 2	7 (35%)	20 (100%)	19.26	<0.001
Pivot Shift Test	Preoperative	Grade 3	13 (65%)	0 (0%)		
Pivot Shift Test	Postoperative (12 months)	Grade 0	17 (85%)	3 (15%)	20.37	<0.001
Pivot Shift Test	Postoperative (12 months)	Grade 1	3 (15%)	11 (55%)		

The comparison of hop test performance between patients with and without LET is presented in Table 4. A higher proportion of patients in Group A achieved successful single-leg hop performance (80% vs 55%); however, a statistical comparison was not performed.

**Table 4:** Hop test performance at 12 months postoperative LET: lateral extra-articular tenodesis

Hop Test	Performance	Group A (With LET), n (%)	Group B (Without LET), n (%)
Single-leg hop test	Able to perform	16 (80%)	11 (55%)
	Unable to perform	4 (20%)	9 (45%)
Double-leg hop test	Able to perform	20 (100%)	18 (90%)
	Unable to perform	0 (0%)	2 (10%)

## Discussion

The effectiveness of LET as an adjunct to ACLR with concomitant lateral meniscal repair was assessed in this study. Although patients were selected based on the presence of rotational instability, the non-randomized, indication-based allocation resulted in baseline differences in the severity of rotational instability between the groups. 

Previous biomechanical studies have demonstrated the important contribution of the lateral meniscus to rotational stability of the ACL-deficient knee. Musahl et al. reported that medial meniscectomy had no significant effect on anterior tibial translation during the pivot-shift test, whereas lateral meniscectomy resulted in a significant increase [17]. Similarly, Hoshino et al. demonstrated using quantitative pivot-shift assessment that concomitant lateral meniscal injury in ACL-deficient knees significantly affects rotational knee laxity [18]. These findings support the importance of addressing both ACL deficiency and associated lateral meniscal pathology when evaluating rotational instability.

Multiple graft types were used in the present study, including hamstring, bone-patellar tendon-bone, and peroneus longus grafts. Graft selection may influence postoperative knee stability and functional outcomes and therefore represents a potential source of variability between groups. Because the study was not designed or powered to assess the independent effect of graft type, this potential confounding factor cannot be excluded and has been acknowledged as a limitation.

The baseline differences between the groups are important when interpreting the comparative outcomes. Patients in the LET group had greater preoperative rotational instability, as reflected by the baseline Lachman and pivot-shift findings. In particular, the higher proportion of high-grade pivot-shift findings in the LET group reflects the indication-based allocation of LET and represents potential confounding by indication. Although the LET group demonstrated greater postoperative rotational stability, these differences cannot be attributed solely to LET because baseline instability and other clinical characteristics differed between groups. Accordingly, the findings should be interpreted as an association rather than definitive evidence of a causal treatment effect.

Despite these baseline differences, the LET group demonstrated greater improvement in postoperative rotational stability, particularly on pivot-shift assessment. At 12 months, 85% of patients in the LET group demonstrated grade 0 pivot shift compared with 15% in the non-LET group. This finding is clinically relevant and is consistent with previous biomechanical and clinical studies demonstrating the role of the anterolateral structures in controlling tibial internal rotation. The contribution of the anterolateral ligament and anterolateral complex to rotational control has been described by Claes et al. [19], while Sonnery-Cottet et al. reported persistent pivot shift following isolated ACLR despite technically successful graft reconstruction [20]. 

The improved rotational stability observed in the LET group is also consistent with previous clinical studies. According to a study by Getgood et al., evaluating the role of LET in ACLR demonstrated a significant reduction in rotational instability and graft failure rates when LET was added to ACLR [21]. Hewison et al. [22] and Jesani and Getgood [23] reported improved rotational control when LET was combined with ACLR. These findings support the potential role of LET in selected patients with increased risk of residual rotational instability. However, given the non-randomized design and baseline imbalance in the present study, the results should be considered supportive rather than definitive evidence of LET efficacy.

The anatomical proximity of the lateral meniscal anterior root to the ACL tibial attachment may present technical challenges during combined ACLR and lateral meniscal repair. The creation of an additional independent tunnel may increase operative complexity and carries a potential risk of tunnel collision, as described by von Essen et al. [24]. De Leissegues et al. reported a reoperation rate of 10% (10/99) in patients undergoing combined ACLR and lateral meniscal repair or transtibial root repair over a mean follow-up of 42 months [25]. Similarly, Anderson et al. reported reoperation in five of 24 patients undergoing combined ACLR and lateral meniscal repair, with a mean follow-up of 58.6 months [26], as also reported by Therrien et al. [27]. These findings highlight the technical considerations associated with combined procedures.

The LET group also demonstrated better performance on the functional hop test. Patterson et al. emphasized the importance of functional recovery following ACLR, reporting an increased risk of progressive patellofemoral bone marrow lesions among patients with poor functional performance one year after ACL reconstruction, defined by a limb symmetry index below 90% on hop testing [28]. Although the present findings suggest a potential association between improved anterolateral stability and functional performance, the results should be interpreted cautiously because functional outcomes may also be influenced by baseline characteristics, graft selection, rehabilitation, and other patient-related factors. Long-term follow-up is required to determine whether these functional differences translate into improved return-to-sport outcomes or reduced reinjury rates.

Both groups demonstrated improvement in anteroposterior stability measures, including the anterior drawer and Lachman tests. This pattern suggests that ACLR provided improvement in sagittal-plane stability in both groups, while the additional benefit associated with LET appeared to be more evident in rotational stability. However, given the baseline differences between groups, these findings should not be interpreted as definitive evidence that LET independently improves rotational stability.

Limitations

This study has several limitations. The small sample size, with 20 patients per group against the calculated requirement of 40 per group, limits statistical power and generalizability. The non-randomized, indication-based allocation resulted in baseline differences in rotational instability and represents potential confounding by indication. Differences in age, sporting activity, and the use of multiple graft types may also have influenced the outcomes. The relatively short 12-month follow-up limits assessment of long-term outcomes such as return to sport, graft failure, and recurrent instability. Finally, outcomes were assessed at multiple time points, but the primary comparative analysis focused on 12-month outcomes. Larger prospective studies with standardized graft selection, balanced baseline characteristics, adequate sample size, and longer follow-up are required to confirm these findings.

## Conclusions

In this cohort of patients undergoing ACLR with lateral meniscal repair, the addition of LET was associated with improved postoperative rotational stability and functional outcomes. However, given the non-randomized allocation, baseline differences in rotational instability, and limited statistical analysis of functional assessments, these findings should be interpreted cautiously. Further adequately powered randomized studies are required to determine the independent effect of LET.
